# Chemical modification of lignin derived from spent coffee grounds for methylene blue adsorption

**DOI:** 10.1038/s41598-020-68047-6

**Published:** 2020-07-06

**Authors:** Fayrouz Taleb, Mohamed Ammar, Mongi ben Mosbah, Ridha ben Salem, Younes Moussaoui

**Affiliations:** 1 Material, Environment and Energy Laboratory (UR14ES26), Faculty of Sciences of Gafsa, University of Gafsa, Gafsa, Tunisia; 2 Organic Chemistry Laboratory (LR17ES08), Faculty of Sciences of Sfax, University of Sfax, Sfax, Tunisia; 3 Faculty of Sciences of Gafsa, University of Gafsa, Gafsa, Tunisia

**Keywords:** Biochemistry, Biotechnology, Materials science

## Abstract

In this work, spent coffee grounds (SCG) were treated using sulfuric acid hydrolysis in order to isolate the sulfuric acid lignin (SAL). The reactivity of SAL was improved through phenolation and acetylation. Spectroscopic analysis showed that the isolated lignin is composed of GHS type and it was characterized by a high amount of (C–C) and β-O-4 bonds. The thermal analysis showed that the phenolated sulfuric acid lignin (Ph-SAL) present higher thermal stability compared to SAL and acetylated sulfuric acid lignin. In addition, the phenolic hydroxyl group content increases from 2.99 to 9.49 mmol/g after phenolation. Moreover, a methylene blue (MB) adsorption test was established in order to find out the sorption capacity of different samples. The study showed that the adsorbed amount of dye increase after the chemical modification of SAL, especially after phenolation. The removal efficiency was enhanced after modification to reach 99.62% for Ph-SAL. The evaluation of the adsorption experimental data with the theoretical models of Langmuir and Freundlich showed that the best fitting was expressed by the Langmuir model for all samples. Finally, this study showed that lignin isolated from SCG can be simply and easily chemical modified and exhibits excellent adsorption ability towards cationic dyes (MB) in aqueous solutions. As a renewable, low-cost, and natural biomass material, lignin from SCG shows a promising practical and economical application of biomass in the field of wastewater purification.

## Introduction

Coffee is one of the most important agricultural products marketed worldwide. Its global production in 2018–2019 has been estimated at 168.87 million bags (1 bag = 60 kg), according to the International Coffee Organization^1^. Nearly 50% of the worldwide coffee production is dealed for soluble coffee preparation, which produced around 6 million tons of SCG per year^2^. Thus; coffee waste represents a high pollution danger if dismissed into the environment unless being exploited for other purposes. However, as this residue is derived from coffee beans, it is expected to have properties similar to these beans and could be thereby exploited for different industrial applications^3^. In this context, some possibilities have been proposed to exploit this coffee residue to be reused in different applications. For example, SCG can be used as substrate for cultivation of microorganisms^4^ and as raw material to produce bioethanol^5,6^ and for the production of biodiesel^7,8^, bio-oil and biochar^9^. Jeguirim et al.^10^ have used the SCG blended with sawdust to produce agropellets for energy production. Chun et al.^11^ have used SCG as polysaccharide-based electro rheological materials. In another study, Ballesteros et al.^12^ developed CMC-based films enriched with SCG extracts containing polysaccharides.

Despite these possible applications, SCG is still slightly used as valuable material. Nowadays, there is a social pressure and great political to minimize the pollution resulting from industrial activities. Therefore, it is needful to exploit SCG, to add value to this unused waste and reduce its impact to the environment. SCG includes great amounts of organic compounds (i.e., lignin, cellulose, hemicellulose, and other polysaccharides…) that can be used as a source of value-added products^13,14^. In particular, Lignin is considered as one of the main components in biomass due to its natural abundance (18–40 wt%)^15,16^. Basically, it is a natural polymer with a complex three-dimensional structure contains wide variety of functional groups including sulfonates, methoxyl, carbonyl, phenolic and aliphatic hydroxyls, and its structure and composition depends on raw material and the isolation method^17,18^. Mostly, Lignin is composed of three phenol derived compounds, guaiacylpropane (G), syringylpropane (S) and p-hydroxyphenylpropane (H)^19,20^. Lignin is inexpensive and possesses numerous interesting belongings, such as favorable rigidity, biodegradability, antioxidant activity, thermal stability and high carbon content^21^. Moreover, lignin has a great potential to act as a precursor for production of bio-fuels and high value chemicals despite its complex structure. The production of high value chemicals from lignin implies the isolation of non-lignin fractions using classical methods such as Klason and other standard methods^22^.

In another hand, different methods of modification have been proposed to insert new chemical active sites onto lignin to enhance its reactivity and physical properties^23^, such as alkylation, hydroxypropylation, esterification, phenolation^24^. Seeing that Lignin is a polyphenol, the functionalization of hydroxyl groups (both aliphatic and phenolic) is a good strategy to develop multiple-functional lignin copolymers. Phenolation of lignin consists of a condensation of phenol with aromatic rings of lignin in acid medium. This reaction can improve its chemical reactivity for synthesizing new lignin-based phenol resins, by introducing more phenolic hydroxyl groups onto lignin^25^. In this context, it was reported that treating lignosulfonate with m-cresol as the phenolating agent and sulfuric acid as the catalyst increase significantly the content of phenolic hydroxyl groups of lignosulfonate^26^. In another research, modifying organosolv lignin using m-cresol improved its physicochemical properties and water solubility^27^. In fact, phenolation is used to enhance the reactivity by increasing the phenolic hydroxyl (Ph–OH) groups so creating more available reactive sites and reduce the molecular weight of lignin, forging it further valuable toward various applications^28^. Acetylation is an esterification reaction that modifies the hydroxyl groups of lignin^29^ involving acidic compounds, acid anhydrides and chlorinated acids as esterification agents^30,31^.

Taking into account information outlined in the writings about the significant amount of Lignin (40%) in the SCG^2,31^, which is elevated than the values mentioned for other lignocellulosic materials such as barley straw (15.50%)^32^, rice straw (17.20%)^33^, sugarcane bagasse (18.93%)^34^, corncob residues (27.01%)^35^, *Astragalus* *armatus* (12%)^36,37^, and *Opuntia* *ficus-indica* (4.8%)^38^. Such information is of great importance to identify the possible area for application of SCG as biowaste. Despite some characteristics of SCG and its application in different fields which have been recently reported in the literature, to the best of our knowledge, there is not yet any study that shows the utilization of SCG for lignin extraction. Considering this fact, the purpose of this study was to recover lignin from SCG using klason method as simple, cheap and fast method, and its chemical modification by phenolation and acetylation, as the simplest procedures of modification that allow its reutilization for further applications, such as dyes sorption.

## Materials and methods

### Chemicals

Sulfuric acid (98%, Sigma Aldrich), phenol (crystallized extra pure, Scharlau), acetic anhydride (99%, sigma Aldrich), pyridine (99.5%, Scharlau), 1, 4-dioxane (99%, Scharlau), methylene blue (99%, scharlau) were used without any further treatment.

Spent coffee grounds was collected from local coffee shops and dried for 1 week in the dark. The residue was washed using distilled water in a Soxhlet apparatus in order to eliminate water soluble extractable then dried in an oven at 105 °C to remove residual moisture.

### Sulfuric acid lignin (SAL) extraction

SAL was prepared from SCG by treating it with 72% sulfuric acid according to the procedure of Klason lignin^39^. The experiment was carried out under magnetic stirring of a mixture of 1 g of washed SCG and 15 ml of the acid solution for 2 h in a water bath at 30 °C. Next a second hydrolysis step was performed using 500 ml of deionized water under reflux at 120 ± 2 °C for 4 h. Finally, the mixture was filtered under vacuum and washed until neutral pH with warm deionized water. The obtained lignin was dried overnight at 50 °C and the yield of SAL was around 27 ± 1%, which is comparable with values found in literature^7^. SAL was grinded in a porcelain mortar and stored in a desiccators before characterization analyses and experiments.

### Chemical modifications of lignin

Phenolation of lignin was performed by dissolving 1 g of SAL and 6 g of phenol in 15 ml of 72% sulfuric acid. The mixture was stirred for 6 h at 60 °C. The suspension was quenched with 560 ml of distilled water and boiled for 3 h. The solid was filtered out with a glass filter, washed with warm distilled water and dried overnight at 105 °C to obtain Ph-SAL.

Acetylation reaction was performed without solvent by using a 1/1 weight ratio of acetic anhydride/pyridine and 1 g of lignin. The mixture was boiled under reflux for 24 h. After precipitation until 100 ml of distilled water, the precipitate was filtered and washed with a mixture of ethanol-distilled water to give Ac-SAL.

## Batch adsorption experiment of MB onto SAL, Ph-SAL, and Ac-SAL

The adsorption experiments were performed at room temperature (25 ± 2 °C) by batch mode as previously described^40–42^. Experiments were carried out by agitating 50 ml of the solution containing the desired quantity of MB, ranging from 10 to 100 mg l^−1^, with 60 mg of the sorbent (SAL, Ph-SAL or Ac-SAL), at the natural pH of the aqueous MB solution, for 24 h. Then, the suspensions were separated from the adsorbent by centrifugation (at 4,000 rpm for 10 min) and filtration through a 0.45 μm membrane filter. MB concentrations were determined spectrophotometrically by detecting the absorbance at λ_max_ = 664 nm using a BECKMAN DU 800. The removal efficiencies and the adsorbed amount of dye ${\mathrm{Q}}_{\mathrm{e}}$ (mg g^−1^) were calculated according to the following equations:

$$Removal\,rate (\%)=\frac{{(C}_{0}-{C}_{e})}{{C}_{0}}\times 100$$

$${Q}_{e}=V\times \frac{{C}_{0}-{C}_{e}}{w}$$

where ${C}_{0}$ and ${C}_{e}$ (mg l^−1^) are the initial and equilibrium concentration of dye solution, respectively. V (L) is the volume of the solution, and $w$ (g) is the weight of the adsorbent.

### Characterization

Elemental analysis was performed on a Thermo Scientific Flash 2000 Organic Elemental Analyzer. C, H, S and N contents were measured and the O content was calculated by difference. The empirical formula was determined from the amount ratio of the main elements C, H, O, N, and S.

Protein content in SAL was estimated by multiplying Nitrogen content by a factor of 6.25, assuming all nitrogen in the sample is from protein^43,44^.

The average double bond equivalent (DBE) relating to the elemental composition (C_a_H_b_N_c_O_d_) was determined using the following equation^45^:

$$DBE=\frac{\left(2a+2\right)-b}{2}$$

where "a" refers to carbon molar ratio and "b" refers to hydrogen molar ratio.

The typical functional groups present in the lignin, Ph-SAL, and Ac-SAL structures were identified by Fourier transform infrared spectroscopy (FT-IR) on a SHIMADZU spectrophotometer. Spectra were recorded within the spectral range between 4,000 and 400 cm^−1^. Each pellet used in the analysis was prepared using 5 mg of the sample to be analyzed and 95 mg of KBr (spectroscopy grade).

The phenolic hydroxyl group contents were determined by the difference UV spectrophotometric method as described by Gärtner et al.^46^ and Nadji et al.^44^, using a BECKMAN COULTER DU 800 UV–visible spectrophotometer.

The ^13^C solid-state nuclear magnetic resonance (NMR) spectrum was employed to analyze both SAL and Ph-SAL structures. Spectra of different samples were recorded using a Bruker AVANCE 400 NMR spectrometer. The NMR probe used was 4 mm with a number of scans of 36,000 for SAL and 3,000 for Ph-SAL. The analysis was performed using cross-polarization technique, with a number of scans of 36,000 for SAL and 3,000 for Ph-SAL and a rotation at a magic angle of 14 kHz.

Thermo-gravimetric analysis (TGA) coupled with differential thermal analysis (DTA) was used to determine the thermal stability, decomposition temperature and char yield for each sample. TGA measurements were taken using Q-50 thermogravimetric analyzer (TA Instruments, USA) setup operating under a nitrogen flow of 50 ml min^−1^ and compressed air with a heating rate of about 10 °C min^−1^. For each measurement, 9 ± 3 mg were used and scans were accomplished from 30 to 800 °C.

The surface morphology of SCG, SAL, and Ph-SAL was performed using FEI ESEM Quanta 200 Electron Microscope. To improve conductivity and quality of image, samples were metalized with a thin layer of Carbon ($\cong$ 10 nm).

## Results and discussion

### Elemental analysis

The elemental analysis shows that carbon, hydrogen, nitrogen and sulfur contents increase in SAL compared to SCG, except the oxygen content which decreases from 42.57 to 25.93% (Table 1). This result is probably due to the decomposition of hydroxyl group and ether bonds in raw material while the extraction of lignin in acidic medium. According to Latif et al.^44^, the presence of nitrogen content SAL, may be due to formation of protein–lignin complex during extraction procedure.

**Table 1:** Elemental composition, DBE, and Ash content (wt%).

Sample	C	H	N	S	O	Ash	DBE
SCG	46.41	6.59	2.51	0.29	42.57	1.62	–
SAL	61.50	8.14	3.72	0.50	25.93	0.21	2.05
Ph-SAL	74.21	8.21	1.21	0.20	16.05	–	3.16
Ac-SAL	63.55	8.23	1.86	0.22	26.10	–	2.18

The presence of protein in SAL (Table 2), suggested that the chemical bond formed between lignin and protein was strong, which resulted in a difficulty to be removed even through acid pretreatment^47,48^.

**Table 2:** Methoxyl and protein content, empirical and C9 formula of SAL**.**

OCH_3_ (%)	Protein (%)	Empirical formula	C9 formula
23.47	23.25	C_5.12_H_8.14_O_1.62_N_0.26_S_0.01_	C_9_H_15.22_O_1.79_(OCH_3_)_1.54_

The double bond equivalent (DBE) for SAL was found to be 2.05. It is related to the degree of condensed lignin and the presence of aromatic ring structure. However this value increases in the case of Ph-SAL (3.16), which confirms the enhancement of aromatic ring in the structure of the modified lignin (Table 1).

The methoxyl content was determined using the predicted formula as published by Jablonsky^49^. The empirical formula and C9 formula of SAL was obtained from the cumulative analysis of all elements as described by Mousavioun and Doherty^50^ and Sameni^51^.

### FTIR spectroscopy

**Figure 1:**
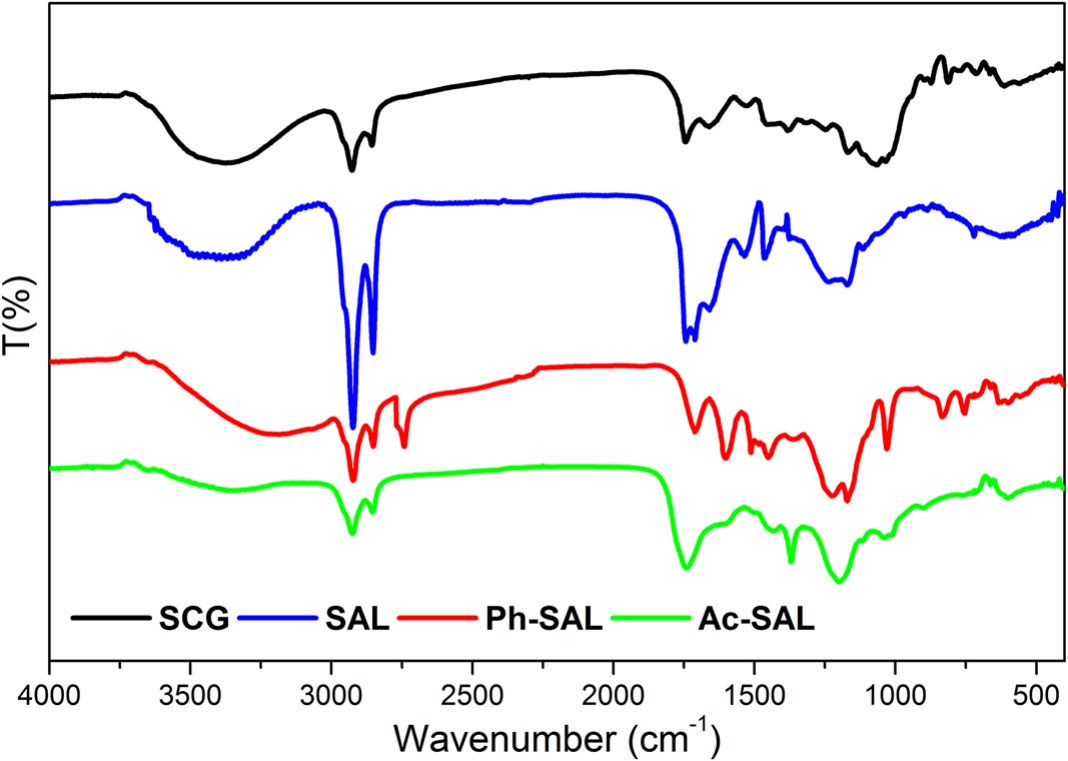
FTIR spectra of SCG, SAL, Ph-SAL, and Ac-SAL.

In this study, FTIR spectroscopy was used to evaluate the chemical bond rearrangement and structural changes of the biowaste before and after klason lignin extraction, as well as changes after lignin modification. FTIR analysis indicates the presence of usual functional groups expected in the lignin structure (Fig. 1).

Figure 1 shows a noticeable shift in peak positions after Klason lignin extraction in the region between 1,000 and 1,250 cm^−1^ for SCG. This clearly shows that Klason lignin extraction has altered the chemical and structural composition of SCG. The small band around 900 cm^−1^ assigned to the glycosidic bond [β-(1 → 4)] in amorphous cellulose was observed in SCG spectra, as well as the bands at 1,375 cm^1^ and 1,473 cm^−1^^11^. However these bands were hardly seen in SAL spectra and similar phenomena was reported in the literature^22^. This shows the efficiency of Klason method towards cellulose and hemicellulose removal to separate pure lignin with much less ash content as shown in elemental analysis results (> 1%)^52,53^. The wide band around 1,000 and 1,250 cm^−1^ in SCG can be attributed to C–OH stretching of polysaccharide in SCG. This band disappears in SAL spectra and a new small peak appears around 1,250 cm^−1^ which correspond to guaiacyl ring breathing with C–O stretching in SAL. Similar results were reported in the literature^22^, pointing out the existence of hemicellulose and cellulose in the region around 1,035–1,200 cm^−1^ and disappearance of these components after different treatment methods.

In SAL spectra, the broad band between 3,100 and 3,800 cm^−1^ was attributed to the hydroxyl groups in phenolic and aliphatic structures. The band at 2,920 cm^−1^ is attributed to C–H stretching in aromatic methoxyl groups and methylene groups of the side chains. However, the band 2,850 cm^−1^ assigned to C-H stretching in the methyl groups. Moreover, the band at 1715 cm^−1^ arises from the non-conjugated carbonyl group stretching. The band at 1,650 cm^−1^ is attributed to the conjugated carbonyl groups in the lignin structure. In addition, the bands at 1,750, 1625, 1531, and 1,500 cm^−1^ in the spectra were assigned to the aromatic skeleton vibrations^53^. Furthermore, the syringyl ring breathing with C–O stretching and guaiacyl ring breathing with C=O stretching also can be seen clearly at the band 1,250 and 1,130 cm^−1^, respectively^22,54^. The weak signal at shoulder around 1,125 cm^−1^ is attributed to the C=O stretching in *p*-coumaric conjugated ester group. This is the typical signal usually observed in HGS lignin^54^. The bands at 1,100 and 1,030 cm^−1^ are assigned to the C–O stretching of the phenolic hydroxyl group and guaiacyl type C–H in-plane deformation, respectively. Moreover, the aromatic C–H out of bending in guaiacyl and syringyl units was showed at band around 820 cm^−1^^40^.

The bands found in SAL’ spectra were similar to that reported by other authors in several analyses of lignin such as, lignins isolated from black liquor^55^, eucalyptus lignins^56^, commercial lignins^57^, olive pomace lignin^58^ and Klason lignin from *P.* *elliottii* sawdust^39^. Nevertheless lignins come from several different sources the functional groups are quite similar to each other. Moreover, the existence of these oxygenated functional groups is consistent with the high percentage of oxygen observed in the elemental analysis results. Since SAL showed a similar behavior to that reported by other authors^55–59^, it is possible to conclude that the isolation procedure of lignin used in this work did not generate a notable change in its chemical structure.

**Figure 2:**
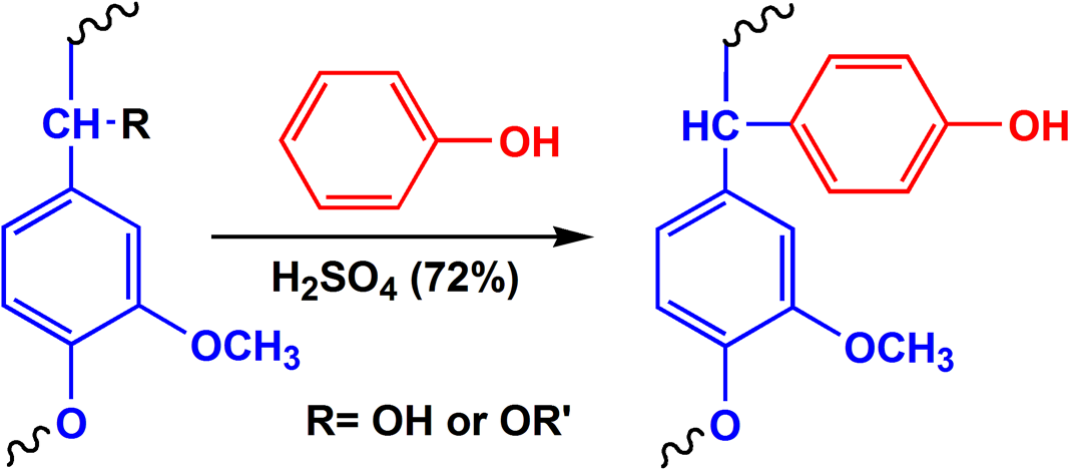
Phenolation process of lignin in acidic medium.

The Ph-SAL spectra shows a broader absorption band to 3,217 cm^−1^ compared with that in the SAL spectra confirming the wealth phenol group of the treated sample. In addition, the appearance of two new bands at 750 and 755 cm^−1^ were found. These bands arise from the reaction between phenol in ortho- or para-position and α-hydroxyl groups in the side chain of lignin in acidic medium as shown in Fig. 2^60^. The disappearance of the band at 1,120 cm^−1^ in the spectra of Ph-SAL is related to ether group’s cleavage during phenolation reaction. Another new intensive band appeared at 2,750 cm^−1^ refers to aromatic C–H stretching in the Ph-SAL as a result of the enhancement of aromatic rings in the SAL structure. Moreover the band at 1,100 cm^−1^ indicating the C–O stretching of the phenolic hydroxyl group become more intensive. In parallel, the two intensive picks around 2,920 cm^−1^ and 2,850 cm^−1^ assigned to C–H stretching in methyl and methylene groups show a noticeable decrease in their intensities.

The acetylation evidence can be found in the relative disappearance of OH absorption band (3,400 cm^−1^), and the appearance of a strong peak in 1,750 cm^−1^, which corresponds to C=O stretching in the carbonyl and carboxyl *p*-substituted due to the introduction of acetyl group. The most significant peak occurs in 1,216 cm^−1^ and characteristic of Ac-SAL (stretching binding C=O, C–C and C–O). Also, the appearance of an intense absorption band in 1,366 cm^−1^, is referring to the C–H bond of elongation in methyl group and phenolic O–H.

### Scanning electron microscopy

**Figure 3:**
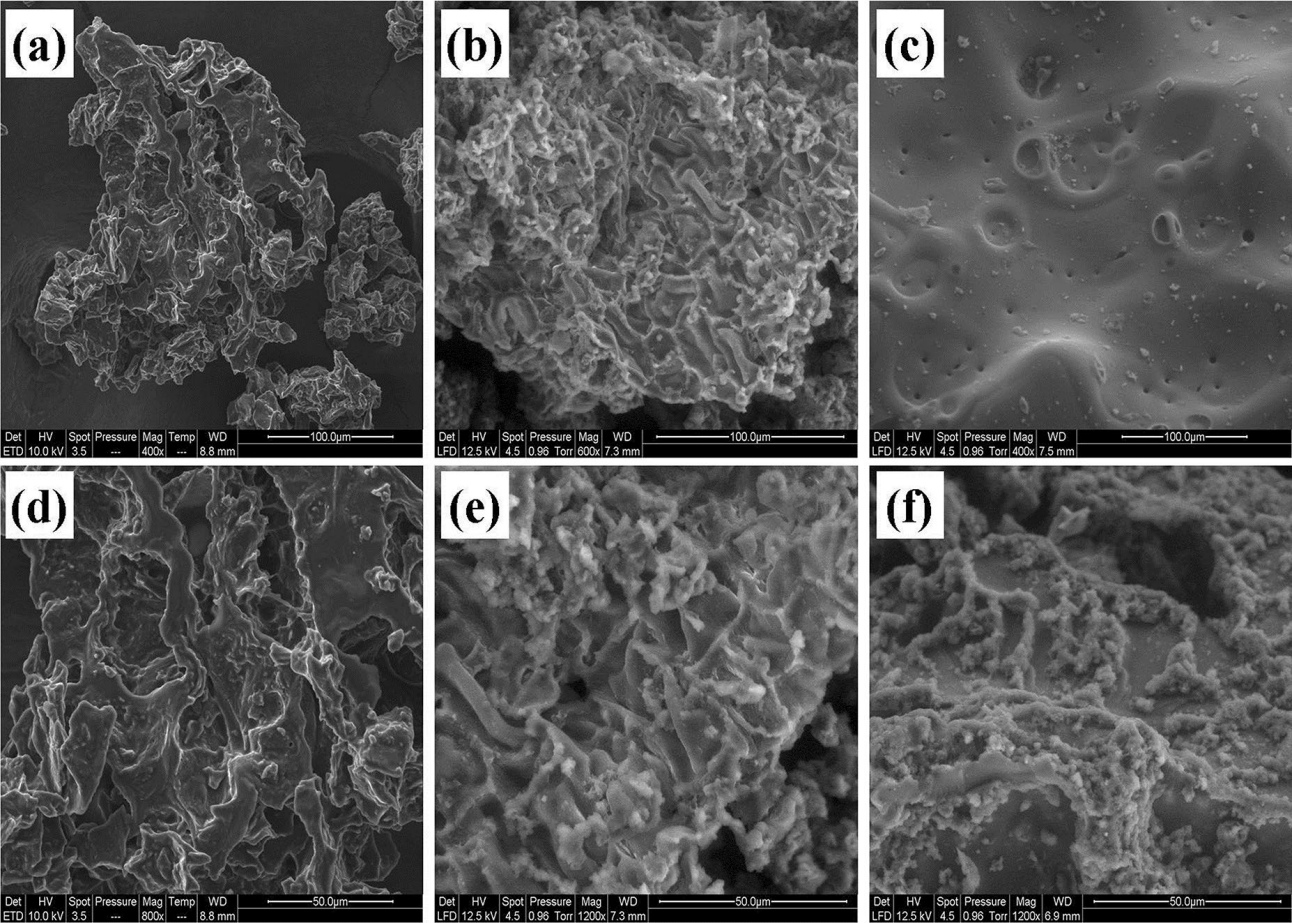
SEM micrographs of SCG (**a**,**d**), SAL (**b**,**e**), and Ph-SAL (**c**,**f**).

SEM images show morphological differences between SCG, SAL, and Ph-SAL (Fig. 3). The micrograph of the untreated (Fig. 3a,d) SCG reveals an uneven surface with cavities of different sizes. Before any chemical modification, the surface of SCG contains wax and other components of biomass. After sulfuric acid treatment, the hydrolysis of cellulose and hemicellulose fraction facilitates the extraction of lignin. The SAL fraction morphology (Fig. 3b,e) presents an irregular shape, and distorted carved structure with cavities. These cavities can be characterized as channels onto the surface of SAL which are useful for dye adsorption. The present study is also in good agreement with the previous reported morphologies of biomass before and after sulfuric acid hydrolysis^22^. After phenolation, the overall morphology seems homogeneous with particles having smooth surfaces. The structure becomes more amorphous with porous surface due to the phenolation in acidic medium.

### Phenolic hydroxyl group content by UV–visible spectroscopy

Considering the lignin as a natural polyphenol with polar hydroxyl sites in its structure^61^, it is necessary to quantify the total phenolic hydroxyl groups. The quantification was determined by the difference UV method, which is based on the difference between absorbance at 300 and 350 nm of free phenolic units in alkaline and neutral solutions^47^. Throw this method it’s obvious to calculate the total hydroxyl group content in SAL and in Ph-SAL to verify the grafting of phenol group using the following equation developed by Gärtner et al.^46^:

$${OH-Ph}_{(tot)}=\left[0.250\times {A}_{300\mathrm{n}\mathrm{m}}\left(NaOH\right)+ 0.107\times {A}_{350}\left(NaOH\right)\right]\times \frac{1}{C\times l}\mathrm{m}\mathrm{m}\mathrm{o}\mathrm{l} \mathrm{g}^{-1}$$

where *A*: absorbance; *C*: to the concentration in g l^−1^; l: path length through the sample in cm.

**Figure 4:**
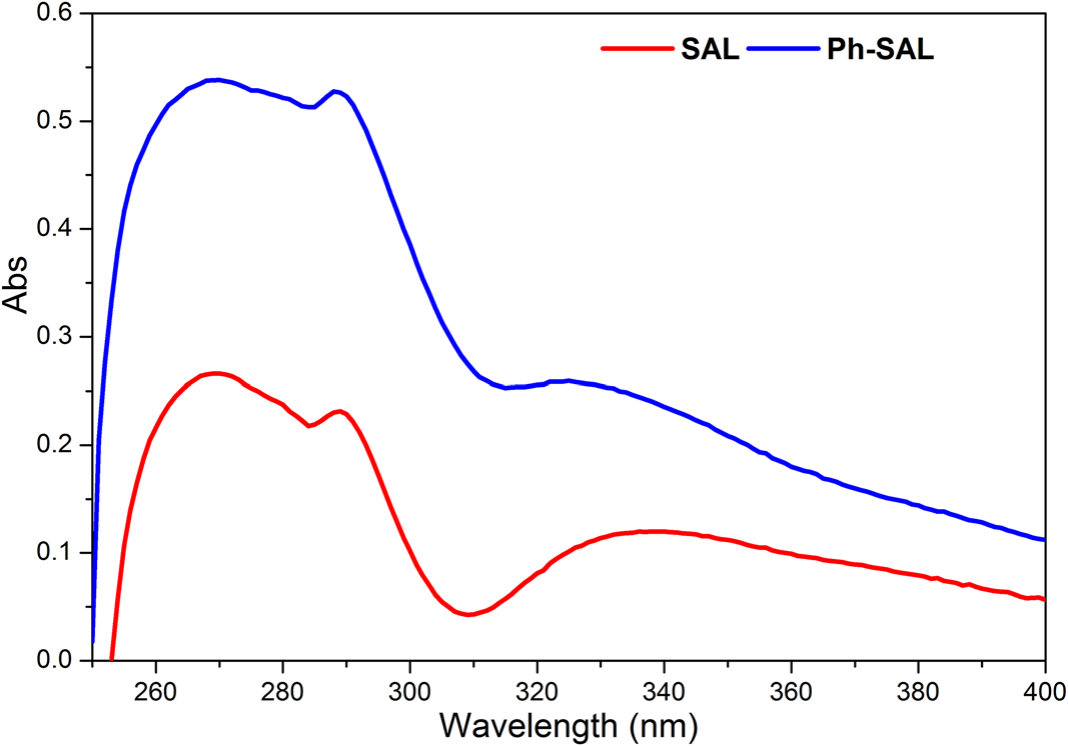
UV spectra of SAL and Ph-SAL.

Both SAL and Ph-SAL have local maximum intensities at 300 and 350 nm which differ in band intensity (Fig. 4). The phenolic hydroxyl content increase from SAL to Ph-SAL (2.99 to 9.49 mmol g^−1^ respectively), proving the enhancement of reactivity of lignin by phenolation process.

### Thermogravimetric analysis

**Figure 5:**
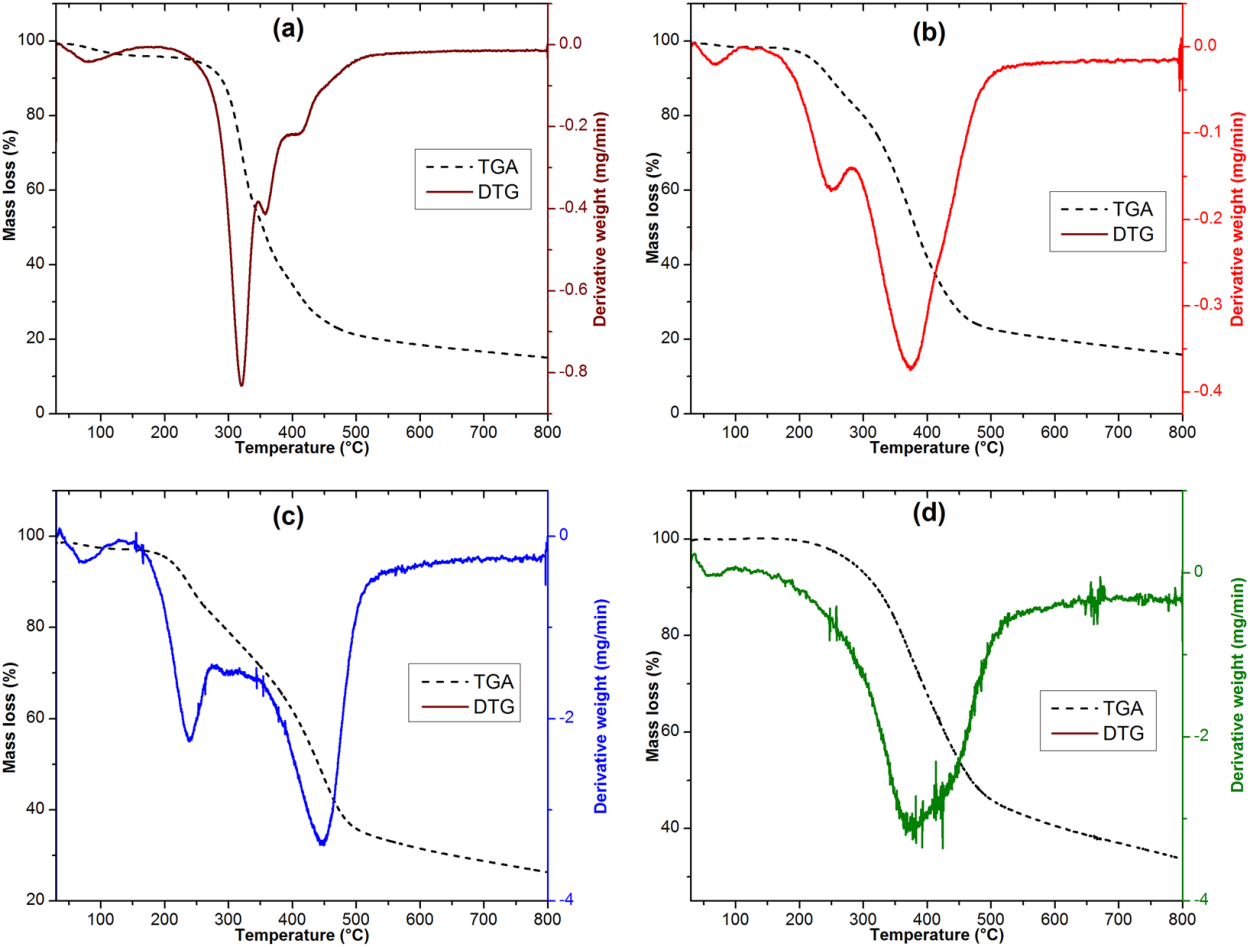
TG and DTG curves of SCG (**a**), SAL (**b**), Ph-SAL (**c**), and Ac-SAL (**d**).

To determine the thermal degradation temperature of the different species contained in each sample thermogravimetric analysis (TGA) coupled to its derivative (DTG) are used (Fig. 5).

Spent coffee grounds present four major weight loss stages (Fig. 5a). The first one starts at approximately 61 °C, and corresponds to soft weight losses of about 2.67% attributed to the release of water (dehydration of the sample). The major transformation occur during the second stage at approximately 228 °C, as a result of easy decomposition of hemicelluloses, which is the most abundant sugar in SCG^3^ and some oils present in SCG takes place, providing weight losses of 38.17%. Cellulose pyrolysis was focused at a higher temperature range (337–378 °C) providing a weight loss of 17.19% attained at 350 °C. The fourth stage is related to the decomposition of lignin, which is the most difficult one to decompose, due to its rich composition of aromatic rings with various branches. This degradation, covering an extremely wide range, occurs in a wide temperature range (378–490 °C) and corresponds to weight loss of about 18.41% attained at 409 °C. The last thermal stage related to the decomposition of the sample starts at about 490 °C with a weight loss of 6.61%^11^.

A weight loss about 1.54% was observed for SAL between 30 and 100 °C (Fig. 5b), attributed to the loss of residual moisture. SAL had a shoulder at about 150 °C with a mass loss around 9.76%, which also slide towards higher temperature, showing that the pyrolysis of lignin is not a single chemical process. This behavior was also encountered in the thermogravimetric curve of the Asian lignin in the research of Jiang et al.^62^. This mass decrease maybe also be attributed to the degradation of components of carbohydrates in the lignin samples, which are converted to volatile gases such as CO, CO_2_, and CH_4_^35,57^.

The main lignin degradation process occurs from 259 to 510 °C, providing a weight loss of 65.35%. The DTG peak is centered at 360 °C. This stage of degradation implies fragmentation of inter-unit linkages and releasing monomeric phenols into the vapor phase that may accelerate the degradation process^63^. Around 20 wt% of SAL samples still remained un-volatized at 800 °C due to the formation of highly condensed aromatic structures which have the ability to form char^64^. Similar results were observed in other lignin samples^39,55,64^.

The thermogram of Ph-SAL (Fig. 5c) shows a dehydration that occurs to about 34.7 °C with mass loss rate of 1.06%. The second thermal degradation started at approximately 153 °C providing a weight loss of 11.31% corresponding to the breaking of weaker chemical bonds in Ph-SAL such as hydroxyl bond, carbon bond in the alkyl side chain, and alkyl ether bond. Above 340 °C, the major weight loss stage of about 38.22% was attained at 430 °C due to the pyrolysis of aromatic rings in Ph-SAL. After phenolation treatment the content of aromatic rings increases^60^, so the modified lignin (Ph-SAL) present higher thermal stability compared to SAL (Table 3). Finally, the remaining matter (8.51%) occurs at 515 °C corresponded to ash, and a constant mass was ultimately obtained.

**Table 3:** Thermal degradation temperatures, DTG peak, and % residue for studied samples.

Sample	Weight loss (%)	DTG peak (°C)	Residue at 800 °C (%)
SCG	38.17	228	6.61
SAL	65.35	360	6.56
Ac-SAL	55.03	350	11.36
Ph-SAL	38.22	430	8.51

The Ac-SAL thermogram (Fig. 5d) show the lowest dehydration (0.05%) comparing to others pyrolysis curves followed by the major weight loss occurs in a wide temperature range (167–511 °C) of about 55.03% attained at 350 °C. This large range of pyrolysis may be attributed to the degradation of lignin polyesters resulting from the esterification reaction which are less stable at low temperature^25^. The charred residue of Ac-SAL at 800 °C was about 11.36%.

In summary, the TGA results (Fig. 5, Table 3) clearly show that the thermal degradation and stability of analyzed samples is different due to the chemical modification on their structure. More importantly, the TGA results show that Ph-SAL is most thermally stable around 430 °C due to its fullness of aromatic rings compared to both SAL and Ac-SAL.

### ***Solid State***^***13***^***C NMR spectroscopy***

**Figure 6:**
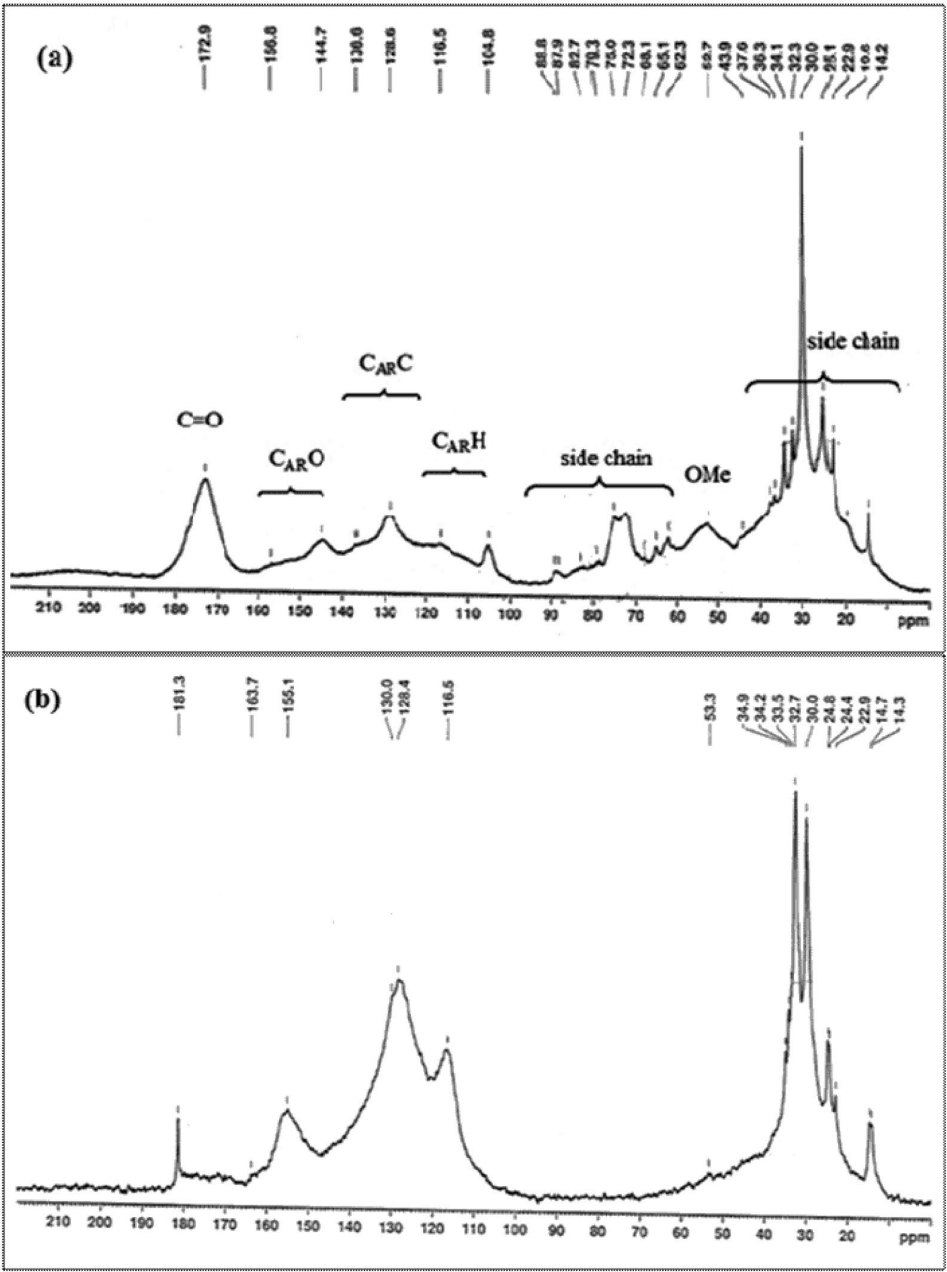
Solid-state ^13^C NMR spectra of SAL (**a**) and Ph-SAL (**b**).

The NMR spectra of SAL (Fig. 6a) show characteristic lignin signals. The OMe carbon was detected at about 54 ppm^53^. Lateral chain carbons of phenyl propane units in SAL are situated between 60 and 85. All noticeable signals were distributed in the region between 100 and 190 ppm for the aromatic carbons. The aromatic region can be divided in three regions as shown in Fig. 6a: protonated carbons C_AR_–H (105–123 ppm), condensed carbons, C_AR_–C (123–147 ppm) which consist of carbons involved in cross-linkages (β–β, β-5…), and oxygenated carbons C_AR_–O (142–160 ppm). In the carbonyl region, a more intense C=O signal assigned to the acetyl carbons appeared at about 172.9 ppm. Syringyl units were identified with the signals between 154 and 152 ppm (C3–C5 etherified) and at 148 ppm (C3–C5 non-etherified). Thus, etherified and non-etherified syringyl aromatic carbons have some degree of overlap with the signals at 144.7 and 136.6 ppm. The lower intensity of the peak at 156.8 ppm SAL can in fact be rationalized by an extensive cleavage of ether function Ar–O–R in Syringyl linkages during acid treatment. This peak decreases more in the spectrum of Ph-SAL (Fig. 6b) and overlaps with the signal at 157 ppm. Guaiacyl (G) units gave a weak signal at 116.5 ppm and the *p*-hydroxyphenyl (H) units appeared at 128.6 ppm (C2–C6). These signals confirmed that the lignin fraction could be assigned as HGS-lignin. These data corroborated by the FT-IR analysis, seeing that the peaks found here are characteristic of the functional groups found in the FT-IR spectrum. Furthermore, the ^13^C-NMR spectra also indicated that β-O-4 is the major linkages in SAL. Thus, three peaks were noticed: at 72.3 ppm refers to C-α, at 62.3 ppm and at 52.7 ppm assigned to C-γ in β-O-4 linkages of guaiacyl and syringyl groups. However, the band at 79.3 ppm assigned to β-1 linkages was detected with much lesser intensity.

The ^13^C NMR spectrum of phenolated lignin (Fig. 6b), show a strongly increasing of intensities of aromatic carbon signals compared to raw lignin due to the attachment of phenol. The integral of the signal at 115 ppm was about twice the integral at 157 ppm, indicating that substitution of phenol in *para*-position was predominating^65^. The methoxyl groups of G and S appears at 53.3 ppm for Ph-SAL. The signals attributed to carbons involved in β-O-4 linkages (C-α and C-γ) for SAL disappear in Ph-SAL which confirm the cleavage of β-O-4 linkages during phenolation in acidic medium. Therefore, Ph-SAL presents a lower amount of (C–C) and β-O-4 bonds, compared to SAL.

## Adsorption efficiency of MB

**Figure 7:**
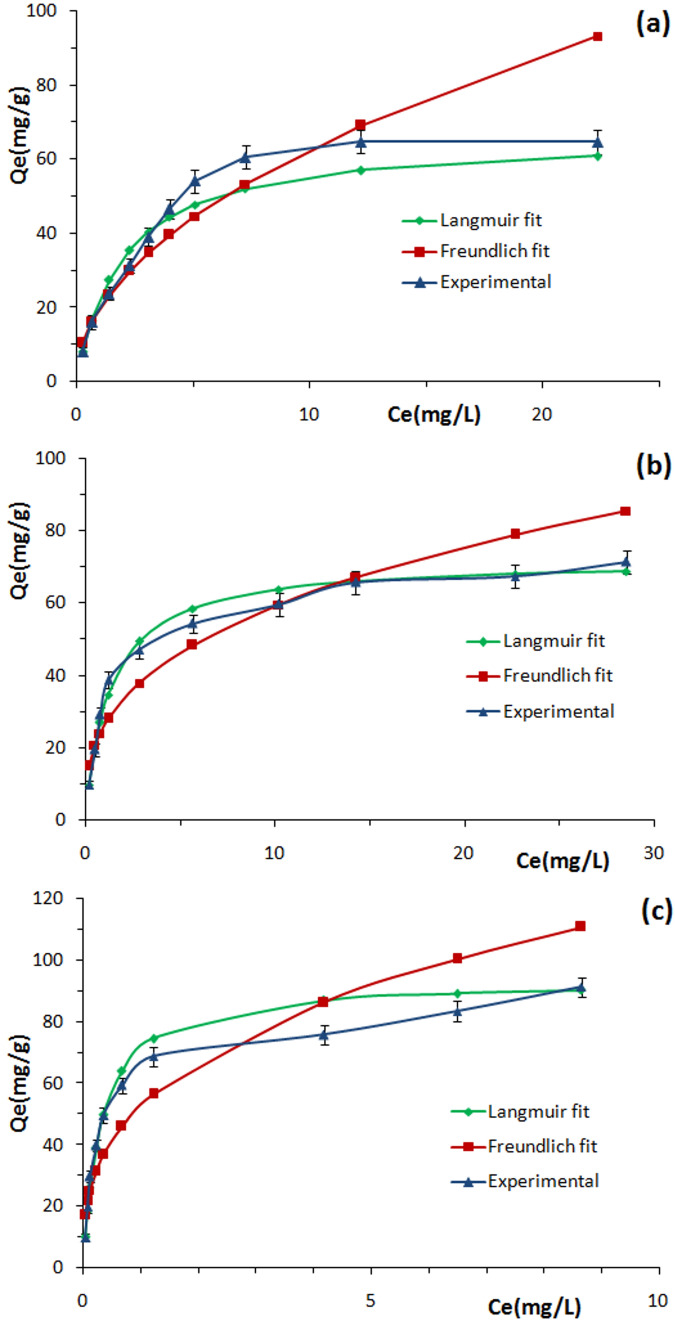
Experimental and modelization isotherm plots for MB sorption onto SAL (**a**) Ac-SAL (**b**), and Ph-SAL (**c**).

The experimental adsorption isotherms obtained for MB were adjusted to the Langmuir and Freundlich models (Fig. 7), for initial concentrations in the range between 10 and 100 mg l^−1^. The results for the obtained parameters are shown in Table 4. Analyzing the values of the linear correlation coefficients (R^2^ is closer to unity), we observed that the experimental data of the adsorption of MB is better adjusted to the Langmuir model for all studied samples. In addition, the *R*_*L*_ values are between 0 and 1; suggest that the adsorption of MB is favorable. As well, the values of 1/n which are lower than unity in Freundlich model, confirm that the sorption was favorable^40^. According to the result of Langmuir analysis, the maximum adsorption capacity of MB onto SAL (*Q*_*max*_ = 66.22 mg g^−1^) consists of a monolayer adsorption and the adsorbent sites are energetically identical^66^. The same observations were noted in the case of Ph-SAL (*Q*_*max*_ = 93.45 mg g^−1^) and Ac-SAL (*Q*_*max*_ = 71.94 mg g^−1^). Many factors would contribute to the adsorption efficiency for the studied samples and the cationic dye; the proposed predominant interactions include hydrogen bonding, electrostatic interactions, and π–π stacking between aromatic rings at the interface between the MB molecules and the sorbent^41,67^. After chemical modification of SAL the adsorption isotherms (Fig. 7) show that the adsorbed amount of MB was enhanced (from 64.69 to 91.34 mg g^−1^ after phenolation, and 71.45 mg g^−1^ after acetylation). In fact, new aromatic rings were grafted after phenolation which may improve π–π interaction between dye molecules and the aromatic region in the Ph-SAL. As well as in the case of Ac-SAL, the electrostatic interactions can be enhanced after creation of new chromophore groups in the modified lignin structure. Thus, improving the amount adsorbed of MB. In addition, the removal efficiency was higher especially in the case of Ph-SAL with a rate of 99.62% compared to Ac-SAL (90.57%) and SAL (77.63%). This result is similar to most other lignocellulosic sorbents mentioned in literatures reported before (Table 5). So we can conclude that the chemical modification of lignin don’t only improves the reactivity of this biopolymer but also, it improves its capacity and its properties as an adsorbent support.

**Table 4:** Langmuir and Freundlich parameters for the adsorption of MB onto SAL, Ac-SAL, and Ph-SAL.

Adsorbent	Freundlich model			Langmuir model			
	*K*_*F*_ (mg g^−1^) (L mg^−1^)^(1/n)^	1/n	R^2^	*K*_*L*_ (L mg^−1^)	*Q*_*max*_ (mg g^−1^)	R^2^	*R*_*L*_
SAL	19.915	0.497	0.930	0.511	66.225	0.993	0.1634–0.0191
Ac-SAL	26.201	0.353	0.879	0.7722	71.942	0.995	0.1146–0.0127
Ph-SAL	52.831	0.343	0.853	3.2424	93.457	0.993	0.0299–0.0030

**Table 5:** Comparison of the adsorbed amount of MB obtained in this study with previous data.

Adsorbent	Initial dye concentration C_0_ (mg l^−1^)	Adsorbed amount of dye Q_e_ (mg g^−1^)	References
Carboxylate-functionalized MSPPS	3,000	1603	^68^
Granular activated carbon from SCG	5,000	986.8	^69^
Organosolv lignin from rice straw	200	20.62	^42^
Activated lignin–chitosan composite extruded blends	82	36.25	^70^
CFBL-silica	100	60	^71^
LBL-silica hybrid		41.6	
SCG	100	23	^72^
Sulfuric acid lignin from SCG (SAL)	100	66.22	This study
Ac-SAL	100	71.94	This study
Ph-SAL	100	93.45	This study

Thereby, SAL and its modified forms had a great potential to be an inexpensive sorbents for cationic dye removal such as MB in wastewater treatment.

## Conclusion

The extraction of sulfuric acid lignin from spent coffee grounds and its characterization was performed, and its modification by phenolation and acetylation process was successfully established and confirmed by spectroscopic analysis. The morphological study shows clearly the change of morphology of the materials before and after processing. Based on the spectroscopy IR and solid state ^13^C NMR, it’s demonstrated that SAL is composed of HGS units. NMR spectroscopy shows that SAL was characterized by a high amount of (C–C) and β-O-4 bonds, compared to Ph-SAL and β-O-4 are the major linkages in the raw lignin macromolecule. Carboxyl fraction shows a decrease in the case of Ph-SAL as a result of phenolation.

The thermal degradation results show that Ph-SAL is most thermally stable around 430 °C due to its fullness of aromatic rings compared to both SAL and Ac-SAL. The enhancement of reactivity of lignin by phenolation process compared to raw lignin was proved by the increase of phenolic hydroxyl content.

We also investigate the potential of different samples as adsorbents to eliminate MB from aqueous solution. The equilibrium data were better described by the Langmuir model and the adsorption ability was favorable for all studied samples. The adsorption amount of MB increase after phenolation and acetylation of SAL, showing a removal rate of more than 90%.

Thus, Ph-SAL presents a promoter material for high potential applications, such as lignin based ion-exchange resin or lignin based phenol resins, which will be expected for future works.
